# Strontium- and Cobalt-Doped Multicomponent Mesoporous Bioactive Glasses (MBGs) for Potential Use in Bone Tissue Engineering Applications

**DOI:** 10.3390/ma13061348

**Published:** 2020-03-16

**Authors:** Farzad Kermani, Sahar Mollazadeh Beidokhti, Francesco Baino, Zahra Gholamzadeh-Virany, Masoud Mozafari, Saeid Kargozar

**Affiliations:** 1 Department of Materials Engineering, Faculty of Engineering, Ferdowsi University of Mashhad (FUM), Azadi Sq., Mashhad 917794-8564, Iran; farzadkermani73@gmail.com (F.K.); mollazadeh.b@um.ac.ir (S.M.B.); 2 Institute of Materials Physics and Engineering, Applied Science and Technology Department, Politecnico di Torino, Corso Duca degli Abruzzi 24, 10129 Torino, Italy; 3 Department of Biology, Faculty of Sciences, Islamic Azad University-Mashhad Branch, Mashhad 917794-8564, Iran; zahragholamzadeh650@gmail.com; 4 Department of Tissue Engineering & Regenerative Medicine, Faculty of Advanced Technologies in Medicine, Iran University of Medical Sciences (IUMS), Tehran 1449614535, Iran; mozafari.masoud@gmail.com; 5 Tissue Engineering Research Group (TERG), Department of Anatomy and Cell Biology, School of Medicine, Mashhad University of Medical Sciences, Mashhad 917794-8564, Iran

**Keywords:** biomaterials, bioglass, mesoporous bioactive glass (MBG), ion release, osteogenesis, bone tissue engineering, bioactivity

## Abstract

Mesoporous bioactive glasses (MBGs) offer suitable platforms for drug/ion delivery in tissue engineering strategies. The main goal of this study was to prepare strontium (Sr)- and cobalt (Co)-doped MBGs; strontium is currently used in the treatment of osteoporosis, and cobalt is known to exhibit pro-angiogenic effects. Sr- and Co-doped mesoporous glasses were synthesized for the first time in a multicomponent silicate system via the sol–gel method by using P123 as a structure-directing agent. The glassy state of the Sr- and Co-doped materials was confirmed by XRD before immersion in SBF, while an apatite-like layer was detected onto the surface of samples post-immersion. The textural characteristics of MBGs were confirmed by nitrogen adsorption/desorption measurements. In vitro experiments including MTT assay, Alizarin red staining, and cell attachment and migration showed the cytocompatibility of all the samples as well as their positive effects on osteoblast-like cell line MG-63. Early experiments with human umbilical vein endothelial cells also suggested the potential of these MBGs in the context of angiogenesis. In conclusion, the prepared materials were bioactive, showed the ability to improve osteoblast cell function in vitro and could be considered as valuable delivery vehicles for therapeutics, like Co^2+^ and Sr^2+^ ions.

## 1. Introduction

There is a long history of successful applications of bioactive glasses (BGs) in the management of bone-related diseases and disorders, as they could improve osteogenesis both in vitro and in vivo [1,2,3,4]. The initial types of BGs were synthesized by using a simple melt-quenching method; however, the high temperatures applied to prepare such glasses (typically around 1500 °C) are considered a critical limitation when incorporation of biomolecules into BGs is a goal to achieve an improvement in biological outcomes [5,6]. On the contrary, sol–gel glasses could be synthesized at lower temperatures and provide better opportunities for drug delivery strategies [7,8,9]. Glasses obtained by conventional sol–gel processes typically exhibit an inherent nanoporosity with quite a broad size distribution, which is not very suitable for drug delivery purposes. Therefore, several attempts have been made to produce glasses with well-controlled nano-sized pores in their structure.

As a special type of sol–gel glasses, mesoporous bioactive glasses (MBGs) have attracted much attention in tissue engineering and regenerative medicine applications. These biomaterials possess favorable textural (ordered pores with size in the range of 2 to 50 nm) and bioactive characteristics and are being used in a wide range of applications, including hard and soft tissue engineering, drug delivery applications, and cancer therapy [10,11,12]. Their suitability in bone tissue engineering for the treatment of osteoporosis has been convincingly documented in a recent animal study [13]. Furthermore, a range of metal ions can be easily incorporated into the glass network during “wet” synthesis processes (sol–gel) [14,15]. In this regard, therapeutic metal ion-doped MBGs are currently under extensive investigation, from the physico-chemical and mechanical properties to biological (in vitro and in vivo) behaviors. For instance, understanding how dopants could affect mesopore size/morphology, surface chemistry, solubility, cell response, and bioactivity of MBGs has become an interesting issue among researchers in the field. On the other hand, the effectiveness of dopants in promoting specific biological properties (e.g., osteogenesis and angiogenesis) should be clarified through well-designed experiments to shed light on the molecular and cellular mechanisms involved in.

It has previously been well documented that strontium is an osteogenesis-inducing element which could be easily substituted with calcium in the BG network [16,17]. Strontium could enhance the bone density through increase in the osteoblast activity and inhibition of osteoclast function (anti-osteoporotic effect) [18,19].

Another element with interesting therapeutic potentials is cobalt. Although commonly considered a toxic element, cobalt could promote new vessel formation (angiogenesis) at concentrations lower than its cytotoxic levels. Hence, the incorporation of cobalt into the BG structure is currently carried out to provide angiogenic activity. The molecular mechanism proposed for the angiogenic effects of cobalt is related to the overexpression of vascular endothelial growth factor (VEGF) and basic fibroblast growth factor (bFGF) (through activation of hypoxia-inducible factor 1-alpha (HIF-1a)), as well as to the enhancement of tubule formation [20,21].

In the present study, we successfully synthesized a series of MBGs doped with strontium (Sr^2+^) and cobalt (Co^2+^) ions, which could be potentially used in bone tissue engineering as multifunctional biomaterials (bioactive, osteogenic, anti-osteoporotic, angiogenic). Our research group previously reported the effectiveness of Sr-and Co-doped melt-derived BGs in vitro and in vivo [17]; MBGs with the same formulations were prepared and investigated here for the first time.

## 2. Materials and Methods

### 2.1. Glass Synthesis

The MBGs were synthesized in a multi-component SiO_2_–P_2_O_5_–CaO–SrO–CoO–Na_2_O-MgO-K_2_O system by using a sol–gel method. All of the reagents were purchased as analytical grade substances (Sigma-Aldrich, St. Louis, MO, USA). The appropriate molar ratio of tetraethoxysilane (TEOS), triethyl phosphate (TEP), Ca(NO_3_)_2_·4H_2_O, Sr(NO_3_)_2_, Co(NO_3_)_2_·6H_2_O, NaNO_3_, Mg(NO_3_)_2_·6H_2_O, and KNO_3_ were defined according to the molar ratio of SiO_2_, P_2_O_5_, CaO, SrO, CoO, Na_2_O, MgO, and K_2_O (Table 1) by using HSC chemistry software (HSC chemistry^®^ for Windows, Outotec, Espo, Finland). In all systems, TEOS was hydrolyzed in the presence of HNO_3_, and then the other reagents were sequentially added in 45 min intervals, maintaining the reaction mixture under constant stirring (first batch, Sol 1). In another batch, 2 g of P123 (EO20-PO70-EO20, M_W_ = 5800 g/mol) was dissolved in 90 mL of ethanol (99.95%) over 60 min (Sol 2). Then, Sol 1 was added to Sol 2 and the mix was stirred for 24 h and stored up in sealed bottles for 7 days (gelation). The gels were aged for 24 h at 60 °C and dried for 24 h in 120 °C. Finally, all samples were heat-treated at 580 °C for 4 h at a rate of 20 °C/min in air.

### 2.2. MBG Characterization

#### 2.2.1. Thermal and Structural Characteristics

Thermal analyses of the gels were carried out by using differential thermal analysis (DTA) (STA 503, BAHR, Germany). Measurements were performed in air with heating rate of 20 °C/min.

The MBG phase compositions were investigated by X-ray diffraction (XRD) (D8-Advance Bruker, Germany) by using monochromatic Cu-Kα radiation (step size 0.01°, time per step 1 s) before and after the incubation in simulated body fluid (SBF). The amount of crystalline/amorphous phases and crystallite sizes of crystalline phases were semi-quantified and evaluated by the Rietveld method by using High Score plus software package (Panalytical Co., for Windows).

#### 2.2.2. FTIR Analysis

The dried MBG powders were analyzed by using Fourier-transform infrared spectroscopy (FTIR) (Thermo Nicolet AVATAR 370, Thermofisher, Waltham, MA, USA) in the range of 400–4000 cm^−1^ to evaluate the major hydroxyl and structural bands in the samples.

#### 2.2.3. Microscopic Observations and Compositional Analysis

The surface morphology and the shape of the synthesized glasses before and after immersion in SBF were characterized by using field-emission scanning electron microscopy (FESEM) (MIRA3, TESCAN, CZ). Compositional analysis was also performed by EDS (included in the FESEM instrument). Transmission electron microscopy (TEM) (CM120 Philips, Amsterdam, The Netherlands) with an accelerating voltage of 100 kV was also employed to investigate more accurately the morphology of as-synthesized MBGs particles.

#### 2.2.4. N_2_ Adsorption–Desorption Analysis

The specific surface area, pore size ranges and pore volume of the MBGs were assessed by nitrogen adsorption/desorption measurements implementing the Brunauer-Emmett-Teller (BET) and Barrett-Joyner-Halenda (BJH) methods (Quantachrome instrument, Anton Paar, Graz, Austria). Before running the tests, the moisture was removed from the MBG samples via the degassing procedure by using a vacuum process at 120 °C for 24 h.

#### 2.2.5. Size and Surface Charge Analyses

Particle size was determined by using DLS analysis (HORIBA Scientific, SZ 100, Kyoto, Japan). Before undergoing particle size assessment, the synthesized MBGs were ground at room temperature in an agate mortar for 10 min. Zeta sizer (Zeta Compact CAD, Les Essarts-le-Roi, France) was used to determine the surface charge of the synthesized MBGs at a constant pH of 7.35–7.45.

### 2.3. In Vitro Bioactivity Assessment

According to the Kokubo’s method, SBF was prepared to evaluate the in vitro bioactivity of the prepared MBGs [23]. Briefly, 0.075 g of glass powders were immersed in 50 mL of SBF (mass-to-liquid ratio = 1.5 mg/mL, as suggested in [24]) and the solution was then shaken with a speed of 20 rpm at the temperature of 37 °C for the time periods of 3 h, 24 h, 3 and 7 days. After the incubation time, the MBGs were observed by using FESEM (MIRA3, TESCAN, CZ). The changes in the pH values of the SBF containing the MBGs were recorded at the time intervals using a universal digital pH-meter (AZ pH Meter 86552, Taichung City, Taiwan). The concentrations of released calcium, phosphorus, silicon, strontium and cobalt were measured by using inductively coupled plasma atomic emission spectroscopy (ICP-OES, Spectro Arcos, Kleve, Germany) at the time points 24 h and 7 days.

### 2.4. Evaluation of In Vitro Cellular Behavior

#### 2.4.1. Cell Viability

The effect of the MBGs on the growth and cell proliferation of osteoblast cells (MG-63 osteosarcoma cell line (National Cell Bank, Pasteur Institute)) was determined by using a standard colorimetric MTT ([3-(4,5-dimethyl-2-thiazolyl)-2,5-diphenyl tetrazolium bromide]) assay (Sigma-Aldrich, St. Louis, MO, USA). For this aim, 1 × 10^4^ cells were seeded in 96-well plates (SPL Lifesciences, Gyeonggi-do, South Korea) and cultured with the RPMI-1640 supplemented by 10% fetal bovine serum (FBS) and 1% penicillin/streptomycin solution (Gibco, Waltham, MA, USA). After 24 h, the culture medium was replaced with a conditioned media containing 4 mg/mL of each type of MBGs. After additional 24 h incubation, the MTT solution (5 mg/mL) was added to the cell culture plates and maintained in a humidified atmosphere of 5% CO_2_ at 37 °C for 4 h. After that, all the media were pulled out and dimethyl sulfoxide (DMSO) solution (Sigma-Aldrich, St. Louis, MO, USA) was added to the plates and shaken for 10 min. Finally, the optical density (OD) of the samples, directly correlated with the viability of cells, were measured by using Synergy H4 Hybrid Multi-Mode Microplate Reader (Synergy HT, BioTek, Winooski, VT, USA) at 570 nm. The experiments were conducted in triplicate, and all of the data were reported as mean ± standard deviation.

#### 2.4.2. Cell Attachment Study

The attachment and expansion of cells onto the material surface are considered as a sign of cyto-compatibility of the substances. Human osteoblastic osteosarcoma cell line (MG-63) (National Cell Bank, Pasteur Institute, Teheran, Iran) were cultured on the MBGs and their morphology was observed by using FESEM (MIRA3, TESCAN, CZ). In brief, all the glass powders were washed with phosphate-buffered saline (PBS) (Gibco, Waltham, MA, USA) three times. Then, 5 × 10^4^ cells were seeded on the samples and incubated for 3 days at 37 °C in a humidified atmosphere of 5% CO_2_. After the incubation, cell-seeded glasses were placed into a solution containing 2.5% (v/v) glutaraldehyde (Merck, Darmstadt, Germany) for 24 h, and the samples were then dehydrated in increasing concentrations of ethanol (Merck, Darmstadt, Germany) (30%, 50%, 75%, and 100%). Before SEM observation, the freeze-dried samples were sputter-coated by gold.

#### 2.4.3. In Vitro Mineralization

Alizarin red staining assay (ARS) was used to determine the bone-like mineralization ability and in vitro osteogenic potential of the MBGs. For this purpose, the osteosarcoma cells at a density of 1 × 10^4^ cells were seeded in 6-well cell culture plates (SPL Lifesciences, Gyeonggi-do, South Korea). The cells were cultured using the conditioned media (4 mg/mL of MBGs) at 37 °C with 5% CO_2_ for a time period of 12 days. At the end of the incubation time, the cells were washed with PBS three times and fixed in 4% paraformaldehyde (Merck, Germany) for 30 min. Cells were then stained by 40 mM alizarin red S solution (pH = 4.2–4.4) (Sigma-Aldrich, USA) for 45 min in the dark at 37 °C. Finally, the cells were washed using distilled water three times. The formed and mineralized calcium layer was viewed by using a high-contrast inverted microscope (Eclipse TS-100, Nikon, Tokyo, Japan). To quantify the results, 25 µL aliquots of each cell culture-well was transferred into a 96-well plate and raised to 100 µL with PBS. The OD at the wavelength of 450 nm was measured by using a microplate reader (Synergy HT, BioTek, Winooski, VT, USA).

#### 2.4.4. Cell Scratch Migration Assay

As a simple and inexpensive test, cell scratch migration assay is used to assess the angiogenic potential of substances in vitro. For performing the assay, 5 × 10^5^ human umbilical vein endothelial cells (HUVECs) (National Cell Bank, Pasteur Institute, Tehran, Iran) were placed into 12-well plates (SPL Lifesciences, Gyeonggi-do, South Korea) and cultured with the Dulbecco’s Modified Eagle Medium/Nutrient Mixture F-12 (DMEM/F-12 (1:1) supplemented by 10% FBS and 1% penicillin/streptomycin solution (Gibco, Waltham, MA, USA) for 24 h. To create a cell-free area, the confluent monolayer was scratched using a 100 µL pipette tip. The culture media were then replaced with the conditioned media (4 mg/mL of each MBGs) and cultured for another 24 h. Finally, the cell migration was viewed by using a high contrast invert microscope (Eclipse TS-100, Nikon, Tokyo, Japan).

### 2.5. Statistical Evaluations

The results of zeta potential values, cell viability, and bone-like mineralization were statistically evaluated by using a two-way analysis of variance (ANOVA). Probability values less than 0.05 were considered significant (* *p* < 0.05, ** *p* < 0.01, *** *p* < 0.001, and **** *p* < 0.0001) (GraphPad Prism, San Diego, CA, USA). Correlation matrix, which shows the correlation coefficient between two variables, was elaborated in R software (R Core Team, version 3.6.1, for Windows).

### 2.6. Image Treatment

The SEM and light microscope images were post-processed (when necessary) by using ImageJ software (NIH, version 1.8.0 for Windows, free software).

## 3. Results

### 3.1. Thermal and Compositional Analysis

**Figure 1:**
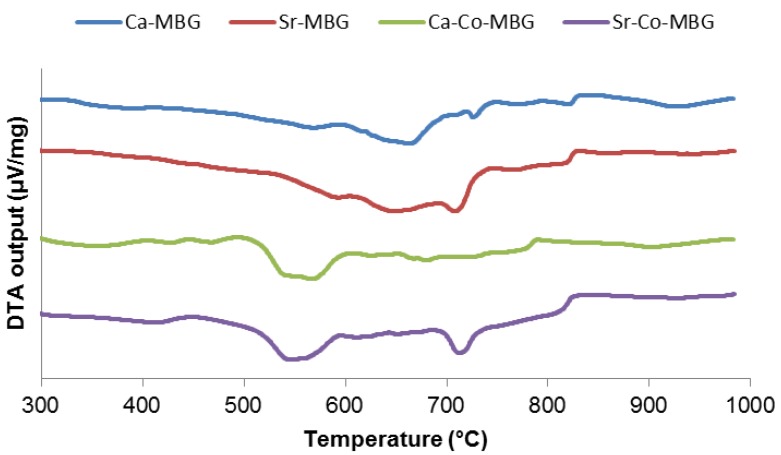
DTA plots resulting from thermal characterization of the gels.

DTA plots of all gels exhibit one or more endothermic peaks between 520 and 720 °C (Figure 1), which can be typically associated with the decomposition of nitrates that were used as precursors of some of the glass oxides. In general, there is a paucity of thermal studies dealing with sol–gel bioactive glasses in the literature and, specifically, the multicomponent systems investigated in this work have never been analyzed previously; therefore, interpretation of DTA thermographs is a challenge. Most of relevant studies focus on the 45S5 composition. Cacciotti et al. [25] reported an endothermic peak associated with the decomposition of nitrates at 529 °C for 45S5-based gel; however, the DTA plot of Ca-MBG gel (i.e., the parent material of our glass batches) revealed a shift of the endothermic peak above 600 °C. This can be attributable both to the higher complexity of the Ca-MBG composition compared to the 45S5 system and to the different heating rate used in the present study for DTA. An endotherm at about 700 °C appears in the Sr-MBG gel, which could be related to the thermal decomposition of strontium nitrate. A broad endothermic peak, which could be attributable to the removal of cobalt nitrate, is visible in the thermograph of Ca-Co-MBG gel. Consistently, both these endothermic peaks can be seen in the DTA plot of Sr-Co-MBG gel, which comprises both strontium and cobalt nitrates in its composition. The small endothermic peaks at around 820 °C (Ca-MBG, Sr-MBG, Sr-Co-MBG) or 780 °C (Ca-Co-MBG) can be attributed to further stages of thermal decomposition of nitrates, especially sodium nitrate, in agreement with Zheng et al. [26].

Compositional analyses performed by EDS (Figure S1, Supplementary Material) confirmed that all the relevant elements of the theoretical compositions have been incorporated in the glass networks.

### 3.2. XRD Analysis

**Figure 2:**
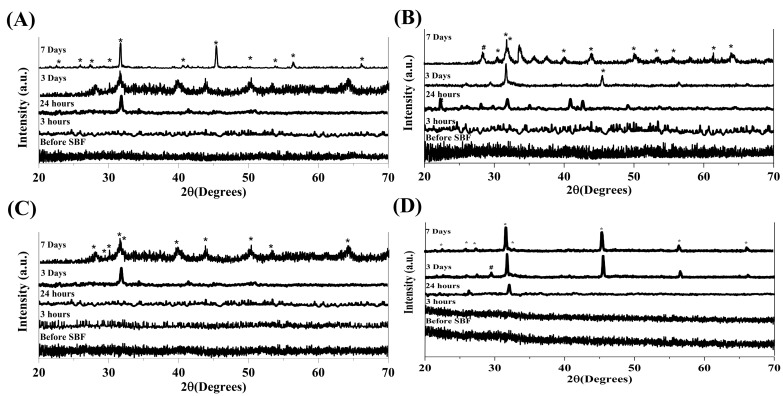
XRD analyses of the synthesized MBGs before and after immersion in SBF for 3 h, 1, 3, and 7 days. Ca-MBG (**A**), Sr-MBG (**B**), Ca-Co-MBG (**C**), and Sr-Co-MBG (**D**). Legend: * Hydroxyapatite (Ca_10_(PO_4_)6(OH)_2_) (Ref. code. 00-009-0432); # Silica gel (SiO_2_) (Ref. code. 01-081-0067).

The XRD results of the glasses before and after the immersion in SBF are shown in Figure 2A–D. Moreover, Rietveld study of the XRD patterns is presented in Table 2, Table 3, Table 4 and Table 5. Rietveld study indicated that the crystallinity of the MBGs is below 5% before soaking in SBF, clarifying the amorphous state of all the samples. The formation of hydroxyapatite (HA) on the glass surfaces was quantified by employing the standard ICDD reference cart of HA (Reference code: 00-009-0432, (Ca_10_(PO_4_)_6_(OH)_2_), Hexagonal, P63m/173). As shown in Table 2, Table 3, Table 4 and Table 5, HA phase can be detected as a main crystalline phase in the SBF-immersed MBGs after 3 and 7 days. According to the data, the crystallinity and crystallite size of the formed HA layer is about 40%–50% and 200 nm, respectively, at 7 days post-immersion in all the samples. According to Rietveld results of crystallinity, it can be stated that an accelerated formation of HCA layer occurred in the doped and co-doped MBGs.

### 3.3. FTIR

**Figure 3:**
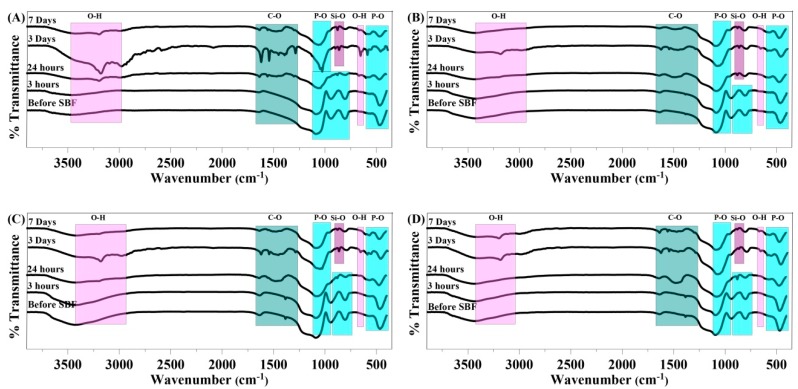
FTIR spectra of the synthesized MBGs before and after immersion in SBF for 3 h, 1 day, 3 days, and 7 days. Ca-MBGs (**A**), Sr-MBG (**B**), Ca-Co-MBG (**C**), and Sr-Co-MBG (**D**).

FTIR spectra of the MBGs are illustrated in Figure 3A–D; the marked bands in the range of (560–630) and (1000–1100) cm^−1^ are assigned to P-O bonds, which are attributed to the presence of HA [27]. Moreover, the observed broad band in the range of (1460–1560) cm^−1^ in all the samples was related to the incorporation of carbonate groups of in the HA layer (hydroxycarbonate apatite (HCA)) [27].

### 3.4. Textural Properties

**Figure 4:**
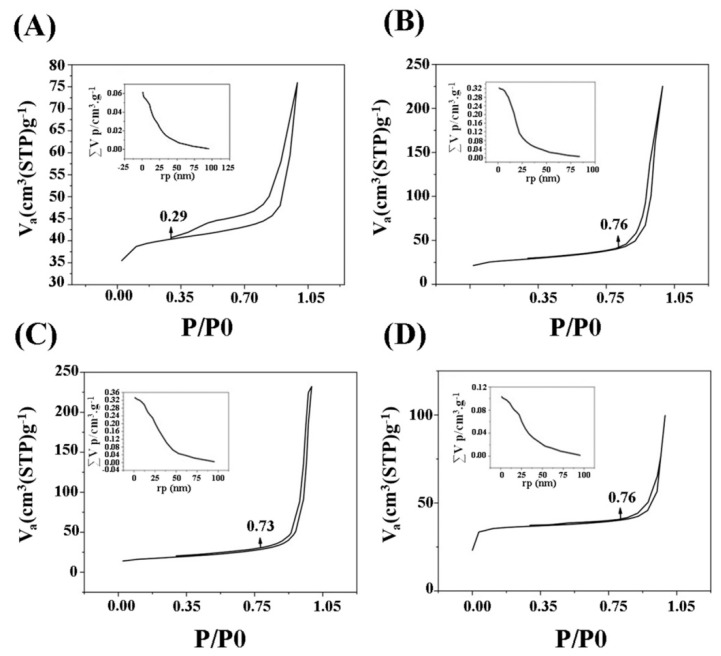
BET/BJH analysis of the synthesized MBGs. Ca-MBG (**A**), Sr-MBG (**B**), Ca-Co-MBG (**C**), and Sr-Co-MBG (**D**).

The results of the BET analysis at 77 K are represented in Figure 4A–D. A hysteresis loop could be clearly observed in all the glass samples. According to the classification provided by the International Union of Pure and Applied Chemistry (IUPAC), the shape of the isotherm in all the glasses can be considered as belonging to the category IV, confirming the mesoporous structure of the samples with a pore size between 2–50 nm. Table 6 collects the textural data of the synthesized MBGs. Ion doping with Sr and Co seems to have an effect in decreasing the specific surface area compared to the parent glass; concurrently, the pore size range moves to higher values of the mesopore size.

### 3.5. DLS, TEM and Zeta Potential Measurements

**Figure 5:**
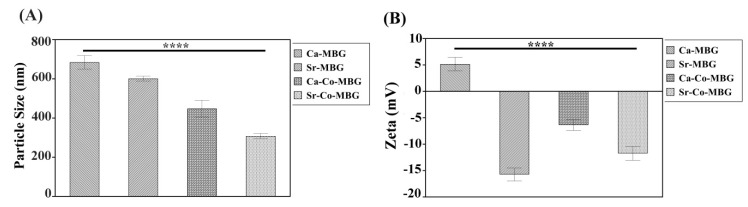
(**A**) DLS and (**B**) zeta potential analysis results.

The values of particle size and zeta potential are displayed in Figure 5A,B; Figure S2 (Supplementary Material) also displays the particle size distributions. Figure 5A shows that the particle size is reduced from 684 to 601, 448, and 308 nm when the glasses are doped with Sr, Co, and Sr-Co, respectively. This reduction is more marked when Ca^2+^ ions (radius: 231 pm) are substituted by ions having a lower ionic radius, i.e., Co^2+^ (radius: 200 pm). As shown in Figure 5B, zeta potential of the MBGs changes from 5 mV for Ca-MBG group to −16, −6, −12 mV for Sr-, Co-, and Sr-Co-MBG groups, respectively, thus suggesting an effect of the dopants on surface charge.

**Figure 6:**
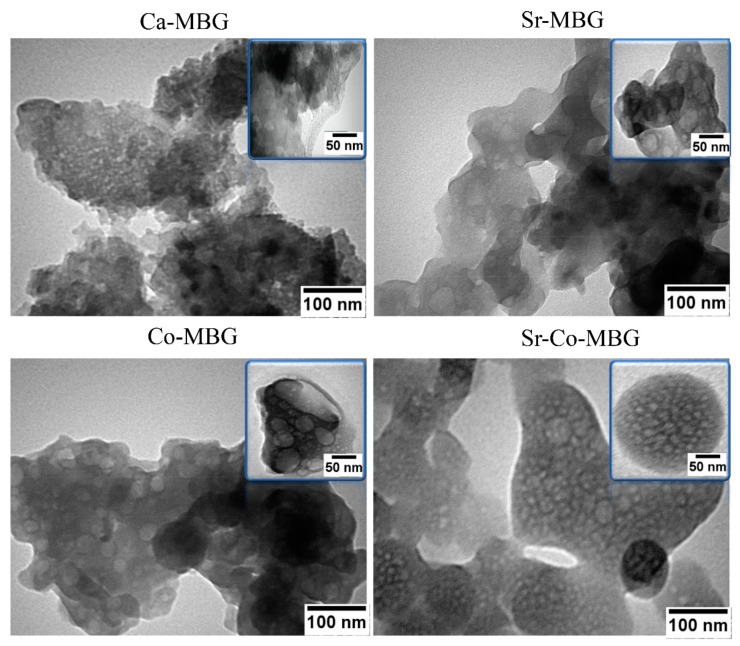
TEM analyses on the as-produced samples.

Figure 6 shows TEM micrographs of the as-synthesized MBGs. The analysis reveals that the particles of all samples have an irregular morphology and a certain tendency to agglomerate, which could explain the apparent discrepancy in the size assessed by TEM (50–100 nm for a single particle) and DLS. TEM investigations also show the presence of intra- and inter-particle porosity in the sample.

### 3.6. Bioactivity

**Figure 7:**
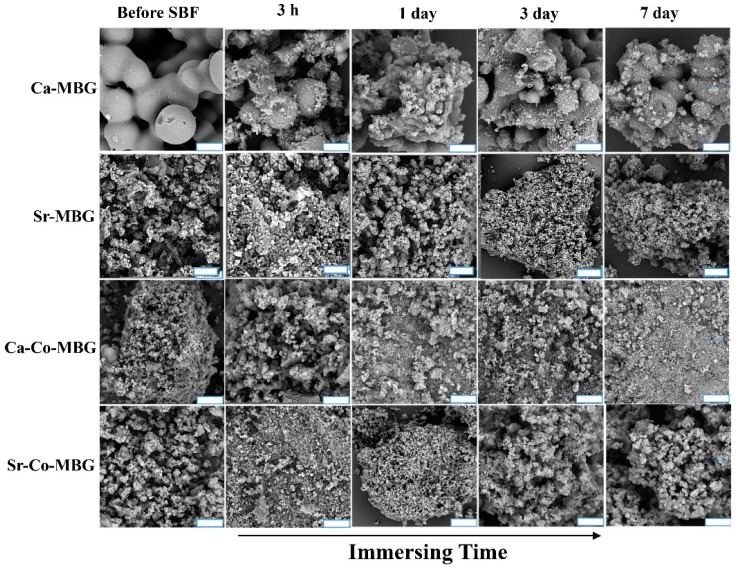
FESEM analysis of immersed MBGs in SBF in periods of 3 h, 1, 3, and 7 days. (Scale bar = 2 µm).

The formation of a HA-like layer onto the SBF-incubated MBGs is confirmed by the results presented in Figure 7. As it can be seen, the formation of a HA-like layer begins at 3 h post-incubation. These results are in accordance with the results of XRD and FTIR analyses.

### 3.7. pH Measurement

**Figure 8:**
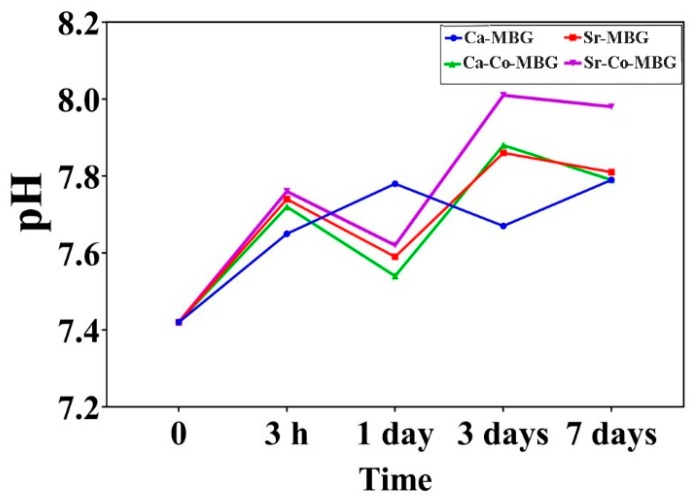
Results of pH measurements in SBF.

The results of pH measurement in the periods of 0, 3 h, 1, 3, and 7 days are shown in Figure 8. The pH values of the SBF increase over incubation time, which is related to the partial dissolution and surface reactivity of the MBGs.

### 3.8. Ion Release Profile

The releases of Ca^2+^, phosphate, silicate, Sr^2+^, and Co^2+^ ions from the MBGs in SBF were measured by an ICP instrument after 24 h and 7 days; the results are reported in Table 7. From a general viewpoint, the release of the various ions from the glasses to SBF is a time-dependent process, i.e., the ion concentration increases with soaking time. Regarding the data, the release of Ca^2+^ from the Sr^2+^-containing MBGs is noticeably more than the other ions, and the highest concentration of the released Ca^2+^ in SBF is about 200 ppm after 7 days of incubation in all the groups. Addition of dopants (Sr and Co) seems to increase the reactivity of the basic material, as suggested by the increased release of silicate ions from Sr-glass, Co-glass and Sr-Co glass especially at 24 h.

### 3.9. Cell Viability

**Figure 9:**
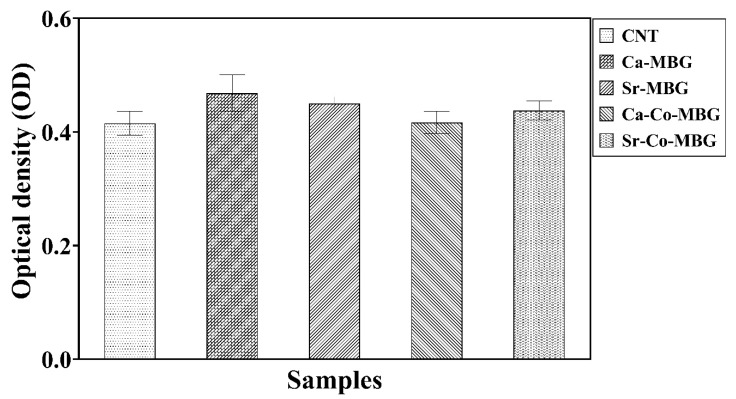
Cell viability: results of MTT assay at 24 h post-incubation.

The results of the MTT assay, which are shown in Figure 9, indicate that none of the synthesized MBGs have any significant adverse effects on the growth and proliferation of MG-63 cells during 24 h post-incubation. No statistically significant differences were found between the Sr/Co-doped glasses and the parent glass as well as between the MBGs and the control (cells cultured without material).

### 3.10. Cell Attachment Study

**Figure 10:**
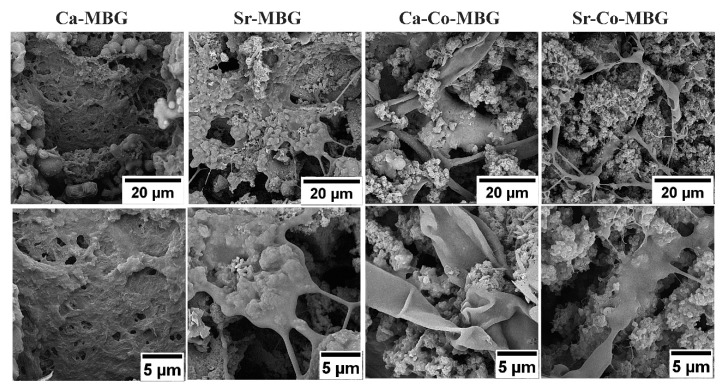
Results of cell attachment study after 3 days.

The SEM micrographs of MBGs seeded with MG-63 cells are shown in Figure 10. The MG-63 cells could well attach and expand (flat morphology) onto the surface of all MBGs.

### 3.11. Bone Nodule Formation

**Figure 11:**
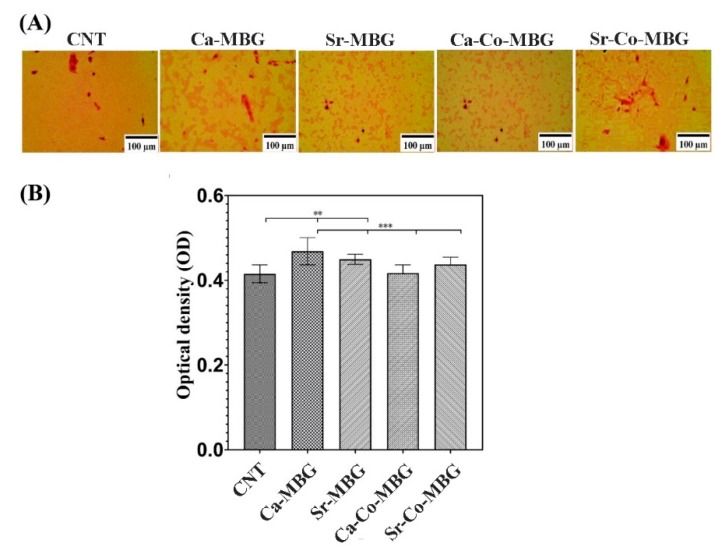
Results of the alizarin red staining assay. High-contrast microscope images (**A**), and quantification results (**B**).

The results of the ARS assay are illustrated in Figure 11A. As can be seen, calcium deposition has occurred in the cells treated with MBGs after 12 days of incubations. Figure 11B shows the quantification results of the ARS-stained samples; the calcium deposition in the culture treated with Ca-MBGs is higher than the other groups.

### 3.12. In Vitro Angiogenesis Evaluation

**Figure 12:**
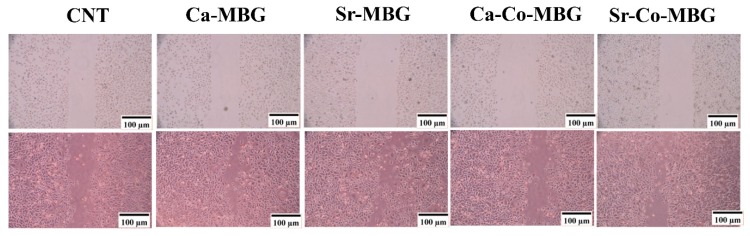
Results of cell migration study (HUVECs) after 24 h.

The optical microscopy images of HUVEC migration upon treatment with MBGs are presented in Figure 12. Based on the obtained results, interestingly, the cell migration is significantly higher in the HUVECs treated with Sr-doped samples in comparison to the other groups.

### 3.13. Correlation Matrix Presentation

**Figure 13:**
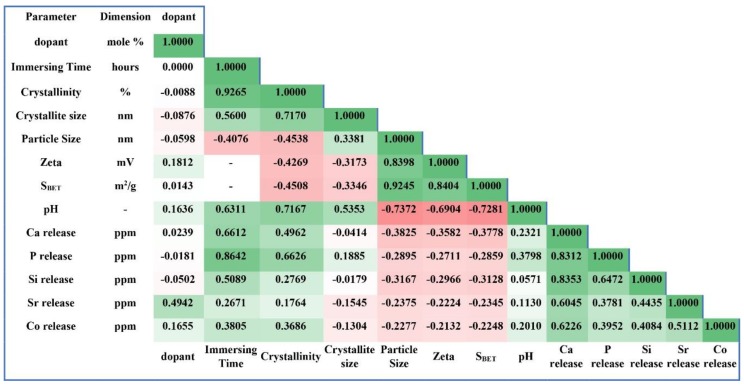
Summary of the acquired data using the correlation matrix.

To summarize the results of the current study, the correlation matrix table of the obtained results was calculated and shown in Figure 13. Each cell in Figure 13 shows the correlation coefficient between two variables (e.g., the crystallite size vs. the amount of dopants). The range of values for the correlation coefficient is −1.0000 to 1.0000. In the correlation matrix, a value of zero indicates that there is no relationship between the two variables. The correlation coefficient higher than zero indicates that there is a positive relationship between variables, and when the value is less than zero, there is a negative relationship. The correlation number of −1.0 indicates a perfect negative correlation, and the correlation number of 1.0 indicates a perfect positive correlation.

## 4. Discussion

The use of MBGs in bone tissue engineering offers several advantages in comparison to traditional melt-derived glasses, including lower synthesis temperature, precise control of the chemistry/composition, and capability to obtain mesoporous particles. In this regard, mesoporous glasses have been proved to be suitable vehicles for the controlled release of drugs, growth factors and therapeutic ions [28]. MBG compositions typically belong to simple basic systems (e.g., the binary SiO_2_-P_2_O_5_ [29] or ternary SiO_2_-CaO-P_2_O_5_ systems [30,31,32]) to which a small amount of the required dopant can be added. The synthesis of complex MBG formulations with multiple co-dopants still is a challenge for biomaterials scientists. In the present study, MBGs in different complex formulations (see Table 1) were synthesized via the sol–gel route by applying the template P123 to create well-ordered pores in their structure. Metal therapeutic ions Sr^2+^ and Co^2+^ were added to MBGs to potentially impart them osteogenic and angiogenic properties.

There are several studies in the literature pointing out that Sr is an apparently safe element in the human body; Neves et al. systematically reviewed Sr-enriched biomaterials and reported them to be effective bone replacements [33]. Recent studies have been addressed to investigating the cardiac safety of Sr-containing formulations, for example, strontium ranelate (SR) [34]. An increased risk for cardiac events has been recorded in patients receiving Sr in randomized controlled trials (RCTs), but this critical situation does not appear in real life [35].

Cobalt is also an essential trace element in the human body and plays key roles as metal constituent in a range of molecules including vitamin B12 and cobalamins [36]. However, high concentrations of this heavy metal may cause severe injuries to various organs like the respiratory, cardiovascular, nervous, and reproductive systems [37]. Experimental data have shown that adverse effects of Co are unlikely to occur at blood doses below 300 µg/L; however, toxic reactions have been detected at lower concentrations in malfunctioning metal-on-metal hip implants [38]. Such Co-associated side effects have been probably related to the persistence of Co^2+^ ions release from permanent metal prostheses in the long term, which does not occur when Co-doped temporary bone substitutes are used.

Burning-off of the polymeric template (P123) during calcination left mesopores in the MBGs, as revealed by TEM results (Figure 6), thus increasing the specific surface area. The mesoporous structure of the un-doped and doped MBGs were confirmed by using BET/BJH analysis and the data are reported in Table 6. A type IV isotherm according to the Brunauer–Deming–Deming–Teller theory (BDDT) classification is observed in all the synthesized glasses. It is stated that the shape of the hysteresis loop (Figure 4) in the obtained graph is closely attributed to the shape of mesopores; specifically, the un-doped and doped MBGs exhibits a H1 hysteresis loop, corresponding to almost well-ordered pores within the structure [39]. The higher amount of surface area along with higher adsorption force are important parameters of a porous material in improving the biological performance (e.g., the gene/drug delivery efficiency). In the case of un-doped MBGs, although the surface area is higher than the doped samples, their adoption force (about 0.3) is significantly lower than that of the doped counterparts (0.7–0.8).

Figure 5 shows the particle size and measured zeta potential of the synthesized MBGs. As displayed in Figure 5A, the glass particle size is reduced after the doping process from 684 to 601, 448, and 308 nm when the glasses are doped with Sr, Co, and Sr-Co, respectively. The presence of the dopant may impede the growth of particles and reduce the particle size. Furthermore, dopants could breakdown the tendency of particles to agglomerate, and thus reduce the particle size. The incorporation of Co^2+^ into the MBGs has significantly reduced the particle size; this was probably connected with the lower radius of Co^2+^ in comparison to Ca^2+^ ions (70 vs. 100 pm, respectively) (see Figure 5A).

The particle size also has an effect on the specific surface area, which can increase due to decreasing the particle size and/or increasing the porosity of particles [40,41]. There is a drop in the specific surface area when Sr or Co are incorporated in the MBGs, which is due to the “disturbing” effect of ions, but when Sr and Co are simultaneously incorporated the material exhibits higher specific surface area (Table 6). This could be explained by considering that Sr-Co-doped MBGs have the lowest particle size (average = 308 nm, Figure 5A) among doped glasses, which thus significantly contributes to increase the specific surface area.

As shown in Figure 5B, zeta potential of the MBGs changes from 5 mV for Ca-MBG group (parent glass) to −16, −6, −12 mV for Sr-, Co-, and Sr-Co-MBG groups, respectively. A similar phenomenon was reported elsewhere for Cu-doped MBGs [42]: the increased zeta potential values may be related to the elevated amounts of positively charged ions in MBGs structure as well as the quick release of the dopants used. It has been previously reported that the surface charge of MBGs have significant effects from a biological viewpoint [43,44,45]. Previous studies dealing with the zeta potential of Sr-substituted materials showed a net negative charge for the particles, which led to an improved biological performance [46]. Therefore, the negative zeta potential created by adding Sr^2+^ and Co^2+^ ions to the MBGs may have a positive effect regarding biological properties.

The XRD results (Figure 2) revealed that all the MBGs are amorphous before immersion in SBF and show bioactive behavior after being soaked in the solution. The peaks related to the formation of a HA layer on the surfaces of all the samples become more visible as the immersion time increases and the incorporation of Sr^2+^ and Co^2+^ had no significant effects on the bioactivity of the glasses. The FTIR spectra of the MBGs confirmed the results obtained from XRD analysis, clarifying the formation of an HCA layer on the sample surfaces at 24 h post-incubation in SBF.

Figure 8 illustrates the pH changes of SBF; an increase in the recorded values is observed for all the MBG groups (from about 7.4 to 7.8 during a 7-day incubation). The pH trend is consistent with the literature, where it is stated that the pH increase happens during the first three days of incubation as a result of the partial dissolution at the glass surfaces due to the high reactivity of these bioactive materials [47]. The interchange between Ca^2+^ from the glass and H_3_O^+^ ions from the solution in the early stages of the bioactivity mechanism is suggested as the main reason of pH increase, facilitating the formation of a HCA layer onto the glass surface.

The release profiles of various glass elements (i.e., Ca, P, Si, Sr, and Co) in SBF were quantified by ICP analysis and are shown in Table 7. The highest release is associated with Ca^2+^ and Sr^2+^ ions. Given the high molar percentage of CaO in all the MBG compositions, high release of Ca^2+^ ions into the surrounding environment is predictable. Moreover, the release of about 70 ppm of Sr^2+^ ions from the Sr-MBG and Sr-Co-groups occurred very quickly (at 24 h in SBF), which could be related to the increasing bond lengths, cation distance, and expansion of the glass network as a result of Sr^2+^ substitution for Ca^2+^. Furthermore, the doping with higher-ionic-radius ions (Sr^2+^) could change the concentration of oxygen vacancies. In this regard, it was reported that transition metal-doped materials could generate and alter the concentration of structural defects, especially oxygen vacancies [48]. Surface oxygen vacancy defects are known to have a substantial effect on the physicochemical properties of materials, such as ions release [48].

Interestingly, the concentration of Sr assessed after 7 days in SBF is lower than that at 7 days. This trend, which contradicts those of all the other elements analyzed, can be explained considering that Sr may be incorporated in the HA-like phase formed on BGs during in vitro bioactivity experiments [49].

The results of MTT assay showed that all the MBGs have no significant adverse effects on the cell viability (Figure 9). As the main components of the BGs are actually trace elements which are naturally found in the human body, BGs are generally identified as cyto-compatible substances in the biological systems. However, rare element-doped glasses should be evaluated in vitro and in vivo as cell behaviors may be affected by such an incorporation. In line with our previous reports on melt-derived BGs [50], the incorporation of Sr^2+^ (6 mol.%) and Co^2+^ (0.5 mol.%) into the MBGs did not inhibit the cell proliferation ability during 24 post-incubation. As shown in Figure 9, the lowest viability value is related to the Ca-Co-MBG (the recorded OD values were 0.437 and 0.461 for the Ca-Co-MBGs and control groups, respectively), which could be attributed to the presence of cobalt in the glass structure.

Following the MTT assay, SEM observations (see Figure 10) confirmed that the cells adhere and grow well onto the MBG surfaces, which further confirm the cyto-compatibility of the synthesized samples. Historically, the application of BGs has been addressed to bone repair and regeneration; in this regard, our silicate-based MBGs show the proper capability of inducing bone nodule formation in vitro (Figure 11A). The quantified measurements revealed that the Ca-MBG group possesses more potential in enhancing in vitro mineralization (the recorded ODs were 0.463 and 0.412 for the Ca-MBG group and control group, respectively). Previously, it has been well demonstrated that in vitro osteogenic potential of BGs can be directly improved by increasing the amount of Ca^2+^ [51].

More recently, special BG compositions have been found to be suitable for promoting additional biological responses, too, such as angiogenesis. In our study, we used the cell scratch migration assay as one of the simplest tests developed to assess angiogenesis in vitro. The mobility of the HUVECs treated with the MBGs showed an increase at 24 h post-incubation (Figure 12). These results are in good agreement with prior reports [20,52,53], and provide early evidence of the ability of Sr/Co-doped MBGs to potentially induce angiogenesis.

## 5. Conclusions

MBGs doped with Sr, Co or both therapeutic elements were synthesized for the first time based on a multicomponent SiO_2_–P_2_O_5_–CaO–Na_2_O-MgO-K_2_O parent system. All the materials exhibited a high specific surface area (>60 m^2^/g), which is key for allowing glass bioactivity. In fact, a fast apatite-forming ability in SBF was observed regardless of the presence of dopants. In vitro cell tests demonstrated the cytocompatibility of the MBGs along with a clear osteogenic potential (calcium nodule formation). Early experiments with HUVECs suggested the pro-angiogenic ability of these Sr-/Co-doped MBGs, which could be therefore proposed as multifunctional systems for advanced tissue engineering applications.

## Figures and Tables

**Table 1:** Nominal compositions (mol.%) of the MBGs produced in this work. The calculations were carried out by using Outotec HSC Chemistry Software [22].

Sample	SiO_2_	P_2_O_5_	CaO	SrO	CoO	Na_2_O	MgO	K_2_O
Ca-MBG	41.20	5.06	36.14	-	-	7.17	3.26	7.17
Sr-MBG	41.20	5.06	30.14	6.00	-	7.17	3.26	7.17
Ca-Co-MBG	41.20	5.06	35.64	-	0.50	7.17	3.26	7.17
Sr-Co-MBG	41.20	5.06	29.64	6.00	0.50	7.17	3.26	7.17

**Table 2:** Summary of the results of Rietveld refinement study for Ca-MBG.

Glass		Rietveld Refinement Study			
Sample	Times	Crystallinity (%)	Hydroxyapatite (wt.%)	Silica gel (wt.%)	Average Crystallite Size (nm)
Ca-MBG	Before SBF	<5	-	-	-
	3 h	8	-	-	-
	24 h	16	-	-	-
	3 days	33.5	97	<5	327 ± 58
	7 days	45	98	<5	198 ± 28

**Table 3:** Summary of the results of Rietveld refinement study for Sr-MBG.

Glass		Rietveld Refinement Study			
Sample	Times	Crystallinity (%)	Hydroxyapatite (wt.%)	Silica gel (wt.%)	Average Crystallite Size (nm)
Sr-MBG	Before SBF	<5	-	-	-
	3 h	9	-	-	-
	24 h	18	-	-	-
	3 days	33.0	98	<5	318 ± 21
	7 days	48	95	5	178 ± 13

**Table 4:** Summary of the results of Rietveld refinement study for Ca-Co-MBG.

Glass		Rietveld Refinement Study			
Sample	Times	Crystallinity (%)	Hydroxyapatite (wt.%)	Silica gel (wt.%)	Average Crystallite Size (nm)
Ca-Co-MBG	Before SBF	<5	-	-	-
	3 h	7	-	-	-
	24 h	16	-	-	-
	3 days	31.0	81	19	316 ± 29
	7 days	46	90	10	201 ± 9

**Table 5:** Summary of the results of Rietveld refinement study for Sr-Co-MBG.

Glass		Rietveld Refinement Study			
Sample	Times	Crystallinity (%)	Hydroxyapatite (wt.%)	Silica gel (wt.%)	Average Crystallite Size (nm)
Sr-Co-MBG	Before SBF	<5	-	-	-
	3 h	<5	-	-	-
	24 h	19	-	-	-
	3 days	28	98	<5	226 ± 9
	7 days	53	89	11	185 ± 24

**Table 6:** Textural characteristics of the synthesized MBGs.

Sample	S_ΒET_ (m^2^.g^−1^)	Range of Pore Radius (nm)	Volume of Pore (V_m_ [cm^3^·g^−1^·(STP)]	P/P_0_
Ca-MBG	151.01	2–10	0.0612	0.29
Sr-Ca-MBG	99.56	5–12	0.3223	0.75
Co-Ca-MBG	62.36	12–23	0.3335	0.70
Sr-Co-MBG	131.97	10–20	0.1033	0.74

**Table 7:** Results of ICP measurements in SBF (ion leaching test).

Sample	Time	Ca	P	Si	Sr	Co
Ca-MBG	24 h	113.45	2.00	7.90	-	-
	7 days	208.81	11.65	31.91	-	-
Sr-MBG	24 h	151.4	1.65	22.53	70.00	-
	7 days	204.70	8.93	30.00	52.00	-
Ca-Co-MBG	24 h	179.569	1.33	22.15	-	0.276
	7 days	193.28	6.47	44.10	-	0.306
Sr-Co-MBG	24 h	175.24	2.23	37.60	69.00	0.432
	7 days	200.80	8.10	41.40	57.00	0.573

## Supplementary Materials

The following are available online at https://www.mdpi.com/1996-1944/13/6/1348/s1, Figure S1: Compositional analyses (EDS) performed on the MBGs after calcination, Figure S2: Particle size distributions assessed by DLS.
