# Supplementary material for: Strontium- and Cobalt-Doped Multicomponent Mesoporous Bioactive Glasses (MBGs) for Potential Use in Bone Tissue Engineering Applications
Source: Materials (Basel). 2020 Mar 16;13(6):1348. doi: 10.3390/ma13061348 (PMC7143072; doi:10.3390/ma13061348)
Supplement: Supplementary file 1 [file materials-13-01348-s001.pdf]

materials

MDPI

Article

Strontium- and Cobalt-Doped Multicomponent Mesoporous Bioactive Glasses (MBGs) for Potential Use in Bone Tissue Engineering Applications

Farzad Kermani [1], Sahar Mollazadeh Beidokhti [1], Francesco Baino [2,*], Zahra Gholamzadeh-Virany [3], Masoud Mozafari [4] and Saeid Kargozar [5,*]

[1] Department of Materials Engineering, Faculty of Engineering, Ferdowsi University of Mashhad (FUM), Azadi Sq., Mashhad 917794-8564 Iran; farzadkermani73@gmail.com (F.K.); mollazadeh.b@um.ac.ir (S.M.B)

[2] Institute of Materials Physics and Engineering, Applied Science and Technology Department, Politecnico di Torino, Corso Duca degli Abruzzi 24, 10129 Torino, Italy

[3] Department of Biology, Faculty of Sciences, Islamic Azad University-Mashhad Branch, Mashhad 917794-8564, Iran; zahragholamzadeh650@gmail.com

[4] Department of Tissue Engineering & Regenerative Medicine, Faculty of Advanced Technologies in Medicine, Iran University of Medical Sciences (IUMS), Tehran 1449614535, Iran; mozafari.masoud@gmail.com

[5] Tissue Engineering Research Group (TERG), Department of Anatomy and Cell Biology, School of Medicine, Mashhad University of Medical Sciences, Mashhad 917794-8564, Iran

* Correspondence: francesco.baino@polito.it (F.B.); Tel.: +39 011 090 4668 (F.B.), kargozarsaeid@gmail.com (S.K.); Tel.: +98-513-800-2539 (S.K.).

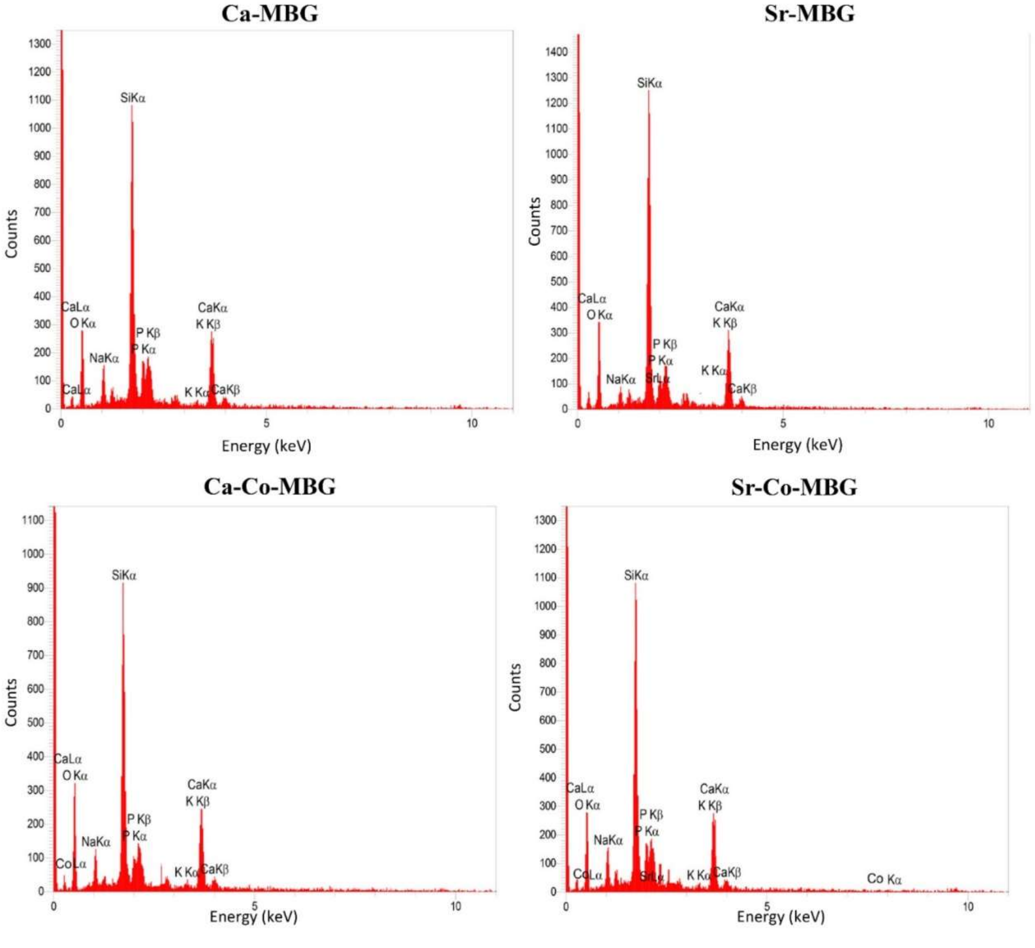

Figure 1. Compositional analyses (EDS) performed on the MBGs after calcination.

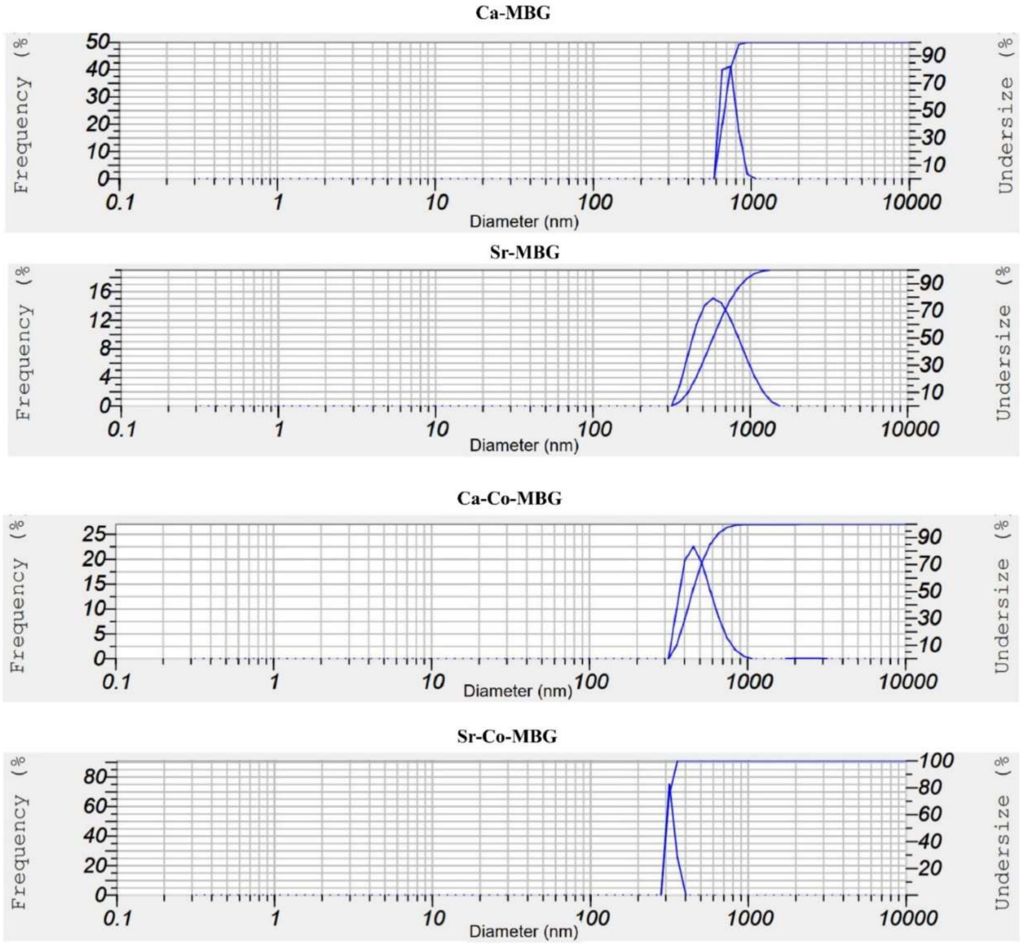

Figure 2. Particle size distributions assessed by DLS.
